# The common G-866A polymorphism of the *UCP2 *gene and survival in diabetic patients following myocardial infarction

**DOI:** 10.1186/1475-2840-8-31

**Published:** 2009-06-15

**Authors:** Barry R Palmer, Courtney L Devereaux, Sukhbir S Dhamrait, Tessa J Mocatta, Anna P Pilbrow, Chris M Frampton, Lorraine Skelton, Tim G Yandle, Christine C Winterbourn, A Mark Richards, Hugh E Montgomery, Vicky A Cameron

**Affiliations:** 1 Christchurch Cardioendocrine Research Group, Department of Medicine, University of Otago, Christchurch, PO Box 4345, Christchurch, New Zealand; 2 Centre for Cardiovascular Genetics, British Heart Foundation Laboratories, Royal Free and University College London Medical School, London WC1E 6JF, UK; 3 Free Radical Research Group, Department of Pathology, University of Otago, Christchurch, PO Box 4345, Christchurch, New Zealand; 4 Current address : University of Denver, 2199 S. University Blvd., Denver, Colorado 80208, USA

## Abstract

**Background:**

A variant in the promoter of the human uncoupling protein 2 (UCP2) gene, the G-866A polymorphism, has been associated with future risk of coronary heart disease events, in those devoid of traditional risk factors and in those suffering from diabetes. We thus examined the impact of the G-866A polymorphism on 5-year survival in a cohort of 901 post-myocardial infarction patients, and the impact of type-2 diabetes on this relationship. The association of *UCP2 *with baseline biochemical and hormonal measurements, including levels of the inflammatory marker myeloperoxidase, was also examined.

**Methods:**

UCP2 G-866A genotypes were determined using a polymerase chain reaction-restriction fragment length polymorphism (PCR-RFLP) protocol. Myeloperoxidase levels were measured in plasma samples taken from 419 cohort patients 24–96 hours after admission.

**Results:**

Genotypes were obtained for 901 patients with genotype frequencies AA 15.5%, GA 45.5%, and GG 39.0%. Genotype was not associated with survival in the overall cohort (mortality: AA 15.6%, GA 16.8%, GG 19.4%, p = 0.541). However, amongst diabetics, AA and GA genotype groups had significantly worse survival than GG diabetic patients (p < 0.05) with an attributable risk of 23.3% and 18.7% for those of AA and GA genotype respectively. Multivariate analysis using a Cox proportional hazards model confirmed that the interaction of diabetes with genotype was significantly predictive of survival (p = 0.031). In the cohort's diabetic subgroup AA/GA patients had higher myeloperoxidase levels than their GG counterparts (GA/AA, n = 51, 63.9 ± 5.23; GG, n = 34, 49.1 ± 3.72 ng/ml, p = 0.041). Further analysis showed that this phenomenon was confined to male patients (GA/AA, n = 36, 64.3 ± 6.23; GG, n = 29, 44.9 ± 3.72 ng/ml, p = 0.015).

**Conclusion:**

Diabetic patients in this post-myocardial infarction cohort with UCP2 -866 AA/GA genotype have poorer survival and higher myeloperoxidase levels than their GG counterparts.

## Background

Uncoupling protein 2 (UCP2) is expressed ubiquitously and is believed to dissipate the proton motive force across the inner mitochondrial membrane [1,2]. While UCP1 may play a role in thermogenesis [3], UCP2 may regulate inflammation and apoptosis, and inhibition of the mitochondrial production of reactive oxygen species (ROS) [4]. These functions have important implications for brain and heart disease. experimental inhibition of *UCP2 *expression increases ROS formation [5,6], a risk factor for atherosclerosis [7]. UCP2 has recently been shown to mediate some of the actions of ghrelin [8], a circulating hormone elevated during fasting and with known actions on the heart [9].

Polymorphisms in the *UCP2 *gene have been associated with obesity [10-12], hypertension [13], and diabetes [14]. A common variant in the promoter of the human *UCP2 *gene (G-866A, rs659366)[11] has been associated with differential *UCP2 *expression [12] and elevated levels of markers of oxidative stress amongst diabetics [15,16]. The A allele has been associated with enhanced *UCP2 *expression in adipose tissue in vivo[11]. Indeed, in a prospective study of 2695 men, those with -866AA genotype exhibited a greater prevalence of obesity and hypertension, and a shorter time to first coronary heart disease (CHD) event. This risk was amplified fourfold in diabetic -866AA men [15].

The enzyme myeloperoxidase (MPO, EC 1.11.1.7) is mainly released by activated neutrophils with pro-oxidative and pro-inflammatory properties. Given the role of inflammation in atherogenesis, MPO is a biomarker of coronary artery disease [17]. Meanwhile, infiltrating macrophages and neutrophils play a role in the destabilization of coronary artery plaques and thus in the pathogenesis of acute coronary syndromes including myocardial infarction (MI)[18].

We thus hypothesized that the *UCP2 *G-866A polymorphism might be associated with survival in a study sample at high risk of future cardiovascular events, and that this relationship might be stronger amongst diabetics. Given the proinflammatory role of UCP2 [4], we also sought association of *UCP2 *genotype with plasma MPO levels in this group.

## Methods

### Study Population

Patients were admitted to Christchurch Hospital between November 1994 and June 2001 and recruited to the Christchurch Post-Myocardial Infarction (PMI) study using criteria described previously [19]. Briefly, MI was defined by typical ischemic symptoms, ischemic change (including ST-elevation or depression or dynamic T-wave changes, i.e. includes ST-elevation, non-ST-elevation, Q-wave, and non-Q-wave infarcts) in two or more electrocardiogram leads and peak elevation of plasma creatine kinase (CK) to at least twice the upper limit of normal. All patients were troponin-T positive. Inclusion criteria included age <80 years, absence of immediate heart failure or cardiogenic shock, and survival for at least 24 h after the onset of symptoms associated with MI. Patients were followed for 5 years and data on mortality/survival for the full 5 years of follow-up was available for all patients in the cohort. Blood samples and cardiac imaging were obtained 24–96 h after symptom onset. Clinical events were determined from recruitment questionnaires, planned follow-up clinic visits, patient notes, the New Zealand National Health Information Service and hospital Patient Management System databases. Ethnicity was self-declared and was grouped as those of European, Maori, Not Stated and Other (Asian, African and Pacific Islanders) ancestry. The investigation conforms to the principles outlined in the Declaration of Helsinki and was approved by the Canterbury Ethics Committee. All participating patients provided written, informed consent.

### Dna Extraction and Genotyping

Genomic DNA was extracted from whole blood as previously described [20]. DNA (100 ng) was amplified for the *UCP2 *G-866A PCR-RFLP assay, in which 360 bp amplimers were digested for 16 h at 37°C using 2.5 U of *Mlu*I [11]. The digested, gel-electrophoresed fragments were visualized using a Bio-Rad Fluor-S™ imaging system.

### Neurohormone, Analyte and Cardiac Imaging Measurements

Circulating levels of endothelin-1, ANP and B-type natriuretic peptide (BNP) and N-terminal pro-BNP (NTproBNP) were assayed as previously described [19]. creatinine clearance was calculated using the Cockcroft-Gault formula [21]. Levels of creatine kinase (CK) were measured using ELISA kits (Roche Diagnostics, Auckland, NZ). Left ventricular function was assessed by radionuclide ventriculography – within 24–96 hours of onset of MI (within 24 hours of blood sampling) [22]. Myeloperoxidase (EC 1.11.1.7) was measured by sandwich ELISA on plasma diluted 1:10, with a monoclonal antibody (Abcam, Cambridge, United Kingdom) and a rabbit polyclonal antibody produced in-house [23], with a detection range of 0.3 to 25 ng/ml and coefficient of variance of 13.8%.

### Statistical Analysis

Univariate analysis, relating *UCP2 *G-866A genotype status to other variables, was performed using χ^2 ^and ANOVA tests. Skewed data (notably plasma hormones) were log-transformed. Survival was assessed using Kaplan-Meier survival curves and log-rank tests. Due to the small number of *UCP2 *-866 AA patients for which MPO levels were available data for AA and GA patients was grouped for this analysis. A Cox proportional hazard model was used to investigate independent risk factors for all-cause mortality. The model included established predictors of prognosis (age, gender, NTproBNP and CK levels, admission left ventricular ejection fraction (LVEF), β-blocker treatment, and creatinine clearance) [20,22,24,25]. Statistical significance was deemed to be achieved at the p < 0.05 level. The statistical power of the study was estimated from the cohort's likely genotype split of ~15% (AA) and ~42.5% (GG or GA respectively)[11,15] with a total n of ~900 and an event (mortality) rate of approximately 3% per annum and complete follow-up to 5 years. Thus there was 80% power to detect >10% difference in mortality between the two homozygote groups, using a two-tailed χ^2 ^test, α = 0.05. All analyses were performed using SPSS version 16.

## Results

### Baseline Characteristics and UCP2 Genotyping

Of an available cohort of 982, genotypes were obtained for 901 patients with genotype frequencies AA 15.5%, GA 45.5%, and GG 39.0% which did not deviate from the Hardy-Weinberg equilibrium (p = 0.999). The minor allele (A) frequency was 0.38, similar to that reported elsewhere [11,15]. Baseline characteristics stratified by *UCP2 *G-866A genotype group are shown in Table 1, and did not differ from those in whom genotyping was not possible. Only patient gender was significantly associated with genotype, with the proportion of males increasing with the number of G alleles. Ethnicity was not significantly associated with differences in genotype frequency (minor allele frequencies: European 0.375, Maori 0.438, Not Stated 0.442, Other 0.371, p = 0.481).

**Table 1:** Baseline characteristics, drug treatment and neurohormonal data stratified by UCP2 genotype group.

	**UCP2 Genotype**						
	**n**	**AA**	**n**	**GA**	**n**	**GG**	***p***
Gender (M/F)	140	102/38(72.9%M)	410	309/101(75.4%M)	351	292/59 (83.2%M)	0.009
Age (years)*	140	61.5 ± 0.94	410	62.0 ± 0.49	350	62.7 ± 057	0.404
BMI* (kgm^-2^)	92	27.2 ± 0.51	285	26.9 ± 0.25	239	26.4 ± 0.25	0.174
LVEF	113	47.2 ± 1.00	348	48.0 ± 0.64	288	47.9 ± 0.72	0.822
Predischarge Mean arterial pressure (mmHg)	133	82.9 ± 0.87	397	83.2 ± 0.55	341	84.6 ± 0.61	0.145
Predischarge Systolic Blood pressure (mmHg)	133	117 ± 1.28	397	116 ± 0.81	341	118 ± 0.90	0.163
Predischarge Diastolic Blood pressure (mmHg)(mmHg)	133	65.9 ± 0.82	397	66.8 ± 0.54	341	67.8 ± 0.57	0.176
*History*							
Previous myocardial infarction*	127	24(18.9%)	389	72 (18.5%)	334	58 (17.4%)	0.896
hypertension *	129	47(36.4%)	400	157 (39.3%)	342	131 (38.3%)	0.847
Type 2 Diabetes*	139	22(15.8%)	409	48(11.7%)	349	38 (10.9%)	0.308
Predischarge Atrial Fibrillation* Fibrillation*	112	12(10.7%)	346	35 (10.1%)	297	47 (15.8%)	0.076
Predischarge Ventricular Fibrillation* Fibrillation*	113	11(9.7%)	355	40 11.3%)	300	21 (7.0%)	0.173
*Laboratory Data*							
Total Cholesterol* (mmol/l)	130	5.94 ± 0.10	376	5.95 ± 0.065	330	5.81 ± 0.068	0.325
HDL Cholesterol* (mmol/l)	129	1.16 ± 0.026	371	1.16 ± 0.017	325	1.15 ± 0.018	0.913
LDL Cholesterol* (mmol/l)	97	3.96 ± 0.10	299	3.98 ± 0.058	268	3.83 ± 0.069	0.225
Triglycerides* (mmol/l)	130	2.02 ± 0.11	376	2.08 ± 0.096	330	2.01 ± 0.069	0.791
Plasma Creatinine† (μmol/l)	136	83 (79–87)	405	85 (82–87)	347	85 (83–87)	0.600
Creatinine Clearance* (ml/s)	99	1.28 ± 0.047	308	1.28 ± 0.023	267	1.32 ± 0.028	0.546
Peak Creatine kinase† (units/l)	105	1440(1250–1660)	327	1610(1480–1740)	269	1640(1510–1680)	0.304
Plasma Glucose (mmol/l)	137	8.55 ± 0.27	404	8.64 ± 0.15	344	8.55 ± 0.16	0.912
Glycated Hemoglobin %	105	6.07 ± 0.11	324	6.06 ± 0.06	274	6.03 ± 0.06	0.905
*Discharge Medications*							
ACE inhibitor *	122	36(29.5%)	374	121 (32.4%)	318	109 (34.3%)	0.624
β-blocker *	122	78(63.9%)	374	232 (62.0%)	318	176 (55.3%)	0.119
Neurohormonal Data							
ANP (pmol/l)‡	138	26.8(24.4–29.4)	403	28.5(27.0–30.0)	346	28.8(27.1–30.6)	0.406
BNP (pmol/l)‡	138	18.2(16.4–20.2)	404	19.8(18.7–21.0)	346	19.9(18.8–21.1)	0.290
NTproBNP (pmol/l)‡	137	98.6(87.0–112)	399	112(104–121)	344	111(103–121)	0.200
Endothelin-1 (pmol/l)‡	137	1.64(1.52–1.77)	398	1.80(1.73–1.88)	340	1.76(1.167–1.81)	0.110

*Means (SEM) or occurrence (percentage); †Median (range); ‡Geometric mean (95% confidence interval).

### Survival and UCP2 Genotype

**Figure 1:**
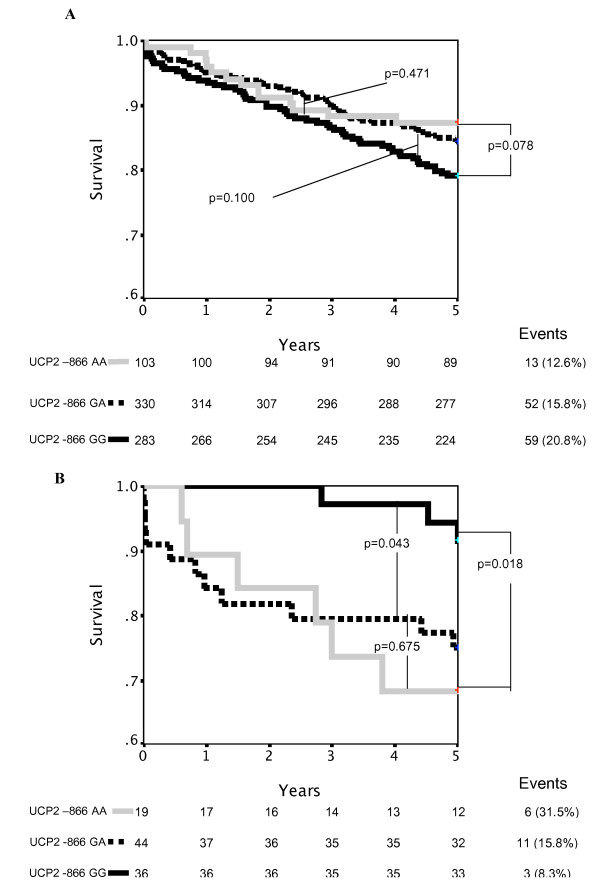
**A Kaplan-Meier survival curve indicating differences in survival after MI for UCP2 G-866A genotype groups in A) non-diabetic patients and B) diabetic patients**.

A total of 159 deaths were recorded in the study cohort during the 5-year follow-up. *UCP2 *G-866A genotype was not associated with survival in the overall cohort (mortality: AA 15.6%, GA 16.8%, GG 19.4%, p = 0.541). However when the cohort was stratified according to prior diagnosis of type-2 diabetes, genotype was significantly associated with survival amongst diabetic patients (Figure 1). Diabetic patients of AA or GA genotype had significantly worse survival than GG diabetic patients (p < 0.05), with an attributable risk of 23.3% and 18.7% respectively. Multivariate analysis using a Cox proportional hazards model confirmed that *UCP2 *G-866A genotype alone was not a significant predictor of survival in the cohort, although there were trends in that direction (Table 2). However the interaction of diabetes with genotype was significantly predictive of survival in a model including age, gender, NTproBNP and CK levels, left ventricular ejection fraction, creatinine clearance, ethnicity, β-blocker treatment, diabetes and genotype (Table 2). Inclusion of additional covariates to the multivariate model did not add significantly to it (e.g. baseline total cholesterol Hazard Ratio = 1.17 [0.96–1.43] p = 0.131; mean arterial pressure HR = 1.01 [0.99–1.04] p = 0.240) and exceeded the recommended number of predictors given the number of events in the analysis.

**Table 2:** Cox's proportional hazards regression model for mortality in the PMI cohort.

	Coefficient	SE	Wald	df	Significance	Hazard Ratio	95% CI for Hazard Ratio
Age	0.018	0.016	1.40	1	0.237	1.02	0.99–1.05
Gender	0.587	0.270	4.71	1	0.030	1.80	1.06–3.06
ACE inhibitor (No/Yes)	0.235	0.229	1.06	1	0.303	1.27	0.81–1.98
LVEF (1–4 days post-MI)	-0.013	0.011	1.48	1	0.224	0.99	0.97–1.01
Log_10 _NTproBNP*	1.55	0.409	14.3	1	<0.001	4.70	2.11–10.5
Log_10 _peak creatine kinase*	1.11	0.338	10.8	1	0.001	3.04	1.56–5.88
Creatinine clearance	-0.900	0.384	5.49	1	0.019	0.406	0.19–0.86
Ethnicity (European v non-European)	1.05	0.597	3.11	1	0.078	2.86	0.89–9.21
Type 2 Diabetes	0.847	0.602	1.98	1	0.159	2.33	0.72–7.59
UCP2 G-866A genotype			4.91	2	0.086		
GG v AA	1.64	0.747	4.79	1	0.029	5.13	1.19–22.2
GG v GA	1.16	0.684	2.89	1	0.089	3.19	0.841–12.2
Diabetes × UCP2 genotype			6.92	2	0.031		
Diabetes × UCP2 genotype(GG v AA)	1.52	0.725	4.40	1	0.036	4.59	1.10–18.9
Diabetes × UCP2 genotype(GG v GA)	2.09	0.826	6.40	1	0.011	8.06	1.60–40.0

* Hazard Ratio represents the change in risk for every 10-fold increase in measurement level.

### UCP2 Genotype and Plasma Myeloperoxidase Levels

**Figure 2:**
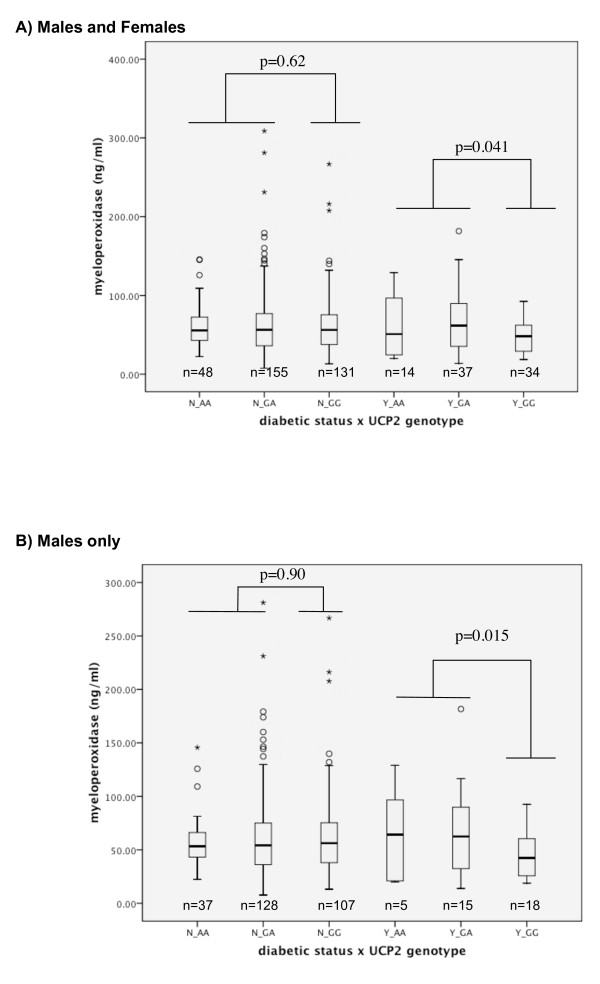
**Plasma myeloperoxidase levels 24–96 hours after index patient admission stratified by UCP2 G-866A genotype and diabetic status a) Male and Female patients, b) Male patients**. N = non-diabetic, Y = diabetic. Stars and circles indicate outlying data.

Plasma MPO levels were available from samples taken from 419 patients in the cohort 24–96 hours after admission. These patients were selected only by the availability of adequate stored plasma for the measurement of MPO and we believe this selection was essentially random. Whilst there was no overall association of *UCP2 *genotype, diabetic status or gender with MPO levels, there was a significant interaction of gender*diabetic status*UCP2 genotype in a univariate general linear model (p = 0.037). In the diabetic subgroup of the cohort, patients with at least one A allele had significantly higher MPO levels than their GG counterparts (GA/AA, n = 51, 63.9 ± 5.23 ng/ml; GG, n = 34, 49.1 ± 3.72 ng/ml, p = 0.041; Figure 2). This phenomenon was only apparent in male patients (GA/AA, n = 36, 64.3 ± 6.23 ng/ml; GG, n = 29, 44.9 ± 3.72 ng/ml, p = 0.015; Figure 2). There was no significant association between genotype and MPO levels in female diabetic patients (GA/AA, n = 15, 62.9 ± 9.99 ng/ml; GG, n = 5, 73.5 ± 6.17 ng/ml, p = 0.561) or amongst non-diabetic patients (data not shown). MPO levels did not differ significantly between diabetic and non-diabetic patients (diabetics, n = 85, 58.0 ± 3.27 ng/ml; non-diabetics, n = 334, 63.7 ± 2.21 ng/ml, p = 0.223).

## Discussion

The major finding of this study is that the interaction between type-2 diabetes and *UCP2 *G-866A genotype was significantly associated with survival in a cohort of post-MI patients. The *UCP2*-866A allele carriers had significantly worse survival during 5 years of follow-up post-MI than GG patients if they were diabetic. These data extend previous observations amongst otherwise healthy men in whom the *UCP2 *G-866A genotype was associated with future risk of CHD events, both in those individuals devoid of traditional risk factors for cardiovascular disease and amongst the small proportion of diabetic patients [15]. In contrast a study of 3122 type-2 diabetic patients reported association of the *UCP2 *-866 A allele with decreased risk of incident coronary artery disease, including sudden cardiac death [26]. The disease-free survival profile in this report closely resembles the survival curve for non-diabetic patients in our study (Figure 1A). This inconsistency may have several explanations: study endpoints differed (survival versus disease-free survival) and differing age and ethnic profiles.

Diabetes is associated with increased oxidative stress [27] and diabetic patients with the *UCP2*-866AA genotype and CHD have been shown to have lower total antioxidant status and higher levels of plasma F2-isoprostanes [15]. Elevated levels of plasma MPO, has previously been shown to be a predictor of mortality in the cohort studied in this report [28]. The difference observed in MPO levels between GA/AA and GG diabetic patients was of similar magnitude to that observed between heart healthy control subjects and PMI patients in the previous report [28]. We did not include MPO as a predictor in the multivariate model of survival as missing data would have severely restricted the number of patients in the analysis. While this data was not well powered to detect differences in MPO levels based on UCP2 genotypes groups, we did detect an association of MPO levels and genotype amongst diabetic patients in this study. This was particularly evident in male diabetic patients in the cohort, a finding consistent with previous reports of gender specific differences in oxidative stress [29,30]. This finding of an association between UCP2 G-866A genotype and plasma MPO levels can be regarded as hypothesis generating and requires validation in other studies.

Although the current study has considerable statistical power, due to the size of the cohort, the length of follow-up, the number of clinical endpoints recorded and the minor allele frequency of the G-866A polymorphism, statistically significant differences may have occurred by chance and weak associations may have been missed. Missing data for some parameters has limited the power of this study to investigate their association with genotype. The study cohort was dominated by ethnic Europeans and the results should not be extrapolated to other populations.

## Conclusion

Our data provide support for the idea that modulation of UCP2 expression might be an important novel target for reducing cardiovascular morbidity and mortality, particularly amongst diabetic patients [31]. A growing body of evidence supports the UCP2 G-866A polymorphism as a marker of cardiovascular risk [15,16,32]. This study extends this evidence to include UCP2 genotype as a potential marker of prognosis in type-2 diabetes patients following acute MI.

## Abbreviations

UCP2: uncoupling protein 2 gene; ROS: reactive oxygen species; MPO: myeloperoxidase; PMI: Post-Myocardial Infarction; BNP: B-type natriuretic peptide; NTproBNP: N-terminal pro-BNP; CK: creatine kinase; LVEF: left ventricular ejection fraction; CHD: coronary heart disease.

## Competing interests

The authors declare that they have no competing interests.

## Authors' contributions

BRP, SSD and HEM conceived the study and the experimental design and BRP, CLD and TJM performed the data acquisition and interpreted the results. TGY was responsible for hormone assay data acquisition and interpretation. BRP, CMF and HEM drafted the manuscript. APP, VAC, AMR, CCW, LS, SSD and HEM interpreted the results and critically reviewed the manuscript adding important intellectual content. All authors read and approved the final version of the manuscript.
